# Internal Phosphorus Loading from the Bottom Sediments of a Dimictic Lake During Its Sustainable Restoration

**DOI:** 10.1007/s11270-018-3937-4

**Published:** 2018-08-11

**Authors:** Katarzyna Kowalczewska-Madura, Renata Dondajewska, Ryszard Gołdyn, Anna Kozak, Beata Messyasz

**Affiliations:** 1 Department of Water Protection, Faculty of Biology, Adam Mickiewicz University, Umultowska 89, 61-614 Poznań, Poland; 2 Department of Hydrobiology, Faculty of Biology, Adam Mickiewicz University, Umultowska 89, 61-614 Poznań, Poland

**Keywords:** Phosphorus, Sustainable restoration, Bottom sediments, Internal loading

## Abstract

The ribbon type Lake Durowskie (Western Poland) is currently undergoing a sustainable restoration process due to water quality deterioration, manifested in water blooms, low transparency, and oxygen deficits near the bottom sediments. Three restoration methods were applied: (i) hypolimnion aeration using two wind-driven pulverizing aerators installed at the deepest places, (ii) phosphorus inactivation using small doses of iron sulfate and magnesium chloride several times a year, and (iii) biomanipulation using pike fry stocking. Research on the exchange of phosphorus in the sediment-water interphase was conducted in the years 2009–2016 to determine the multiannual changes of internal phosphorus loading from bottom sediments during the restoration process. The sustainable approach resulted in a decrease of internal phosphorus loading and a gradual increase in the sorption capacity of bottom sediments, particularly noticeable in the last 2 years. The content of phosphorus in the sediment and in the interstitial water fluctuated, showing an increase during the first years of restoration and then a gradual decrease. It was proved that the process of sustainable restoration is cheap and does not interfere strongly with the ecosystem, although it is long-lasting. It should be continued for many years, especially in the case of continuous external loading of the lake with nutrients from the catchment area, i.e., until the water quality in the main tributary improves and the lake ecosystem stabilizes.

## Introduction

Lake water quality is influenced by many factors, whose source is both in the catchment area (external loading) and in the aquatic ecosystem (internal loading). Nutrients, phosphorus (P) in particular, have been considered the most important factor influencing lake water quality. It is a key element in controlling primary production in lakes (Kuczyńska-Kippen and Joniak 2010; Søndergaard et al. 2001, 2017) and most of it is accumulated in lake sediments, from which it returns to water column mainly as orthophosphates. Particulate and organic P forms may reload the water column for a short time, e.g., after extensive sediment resuspension events (Markou et al. 2007). The net release of P observed from a sediment is the difference between the sedimentation of particles (algae, detritus) and phosphorus adsorption on bottom sediments on the one hand and the release of phosphorus into the water column on the other (Søndergaard et al. 2001). Internal loading caused by P released from anoxic sediments often represents the main summer P load to lakes and can have an immense effect on their water quality, especially the eutrophication process (Lennox 1984; Søndergaard et al. 2001; Nürnberg 2009). The release of P from bottom sediments is influenced by many factors: temperature, oxygen concentration and gradient of P concentration in the sediment-water interphase, pH, redox potential, presence of macrophytes, type of chemical compounds in which phosphorus is present in the sediments, and the structure of the sediments (Stephen et al. 1997; Søndergaard et al. 2001, 2002; Golterman 2004). Nowadays, many methods are used for reducing internal P loading (Søndergaard et al. 2001; Zamparas and Zacharias 2014; Bormans et al. 2015). These are physical (aeration, oxygenation), chemical (increasing sediment sorption capacity by adding iron, aluminum, and calcium), and biological methods (biomanipulation). Recently, a frequent approach has been to use several methods of lake restoration simultaneously, in a sustainable way (Pan et al. 2012; Gołdyn et al. 2014). In trying to exploit natural mechanisms of self-regulation, we make the ecosystem less prone to serious disturbance. Most data relating to the internal loading of lakes concerns shallow water bodies (e.g., Kozerski and Kleeberg 1998; Søndergaard et al. 2001; Dondajewska 2008; Kowalczewska-Madura and Gołdyn 2009; Joniak and Kuczyńska-Kippen 2010; Kowalczewska-Madura et al. 2010), but little is known about the release of phosphorus from deep, dimictic lakes (e.g., Kleeberg 1997; Murphy et al. 2001), especially those subjected to restoration treatments (Kowalczewska-Madura et al. 2015).

Therefore, the main aim of the present study was to determine the influence of long-term restoration with the use of three sustainable restoration methods on the intensity as well as seasonality of P internal loading from the bottom sediments of the strongly eutrophicated Lake Durowskie.

## Study Area

Lake Durowskie is a ribbon type postglacial, eutrophic, urban lake situated in Wągrowiec, 60 km from Poznań (Wielkopolska Region, West Poland). Its coordinates are 17° 12′ 1″ E and 52° 49′ 6″ N. Its surface area is 143 ha and maximum depth is 14.6 m (Table 1). It is elongated in shape (oriented north-south) and the Struga Gołaniecka River flows along its longitudinal axis. A cascade of hypereutrophic lakes is situated in the river course above Lake Durowskie. These are Kobyleckie, Grylewskie, Bukowieckie, and Laskownickie lakes, with cyanobacterial water blooms in summer.

**Table 1:** Morphometric data of the Durowskie Lake (Jańczak 1996)

Parameter	Value
Total area	143 ha
Volume	11,322,900 m^3^
Mean depth	7.9 m
Maximum depth	14.6 m
Maximum length	4340 m
Maximum width	540 m
Length of the coastline	10,515 m

Lake Durowskie is used for recreation and both economic and sport fishing activities not only for residents of the town but also for visitors from the surrounding areas. For this reason, it is important to maintain a good ecological status of the lake according to the requirements of the Water Framework Directive (WFD 2000). More pressure is exerted on the deeper, southern part of the lake, adjacent to the city of Wągrowiec. Apart from urbanization, the recreation and touristic activity have a significant impact here. During the 1990s, charophytes could still be observed in lake, mainly *Charetum tomentosae* and *Charetum contrariae* (Nagengast 1998).

Progressive eutrophication of Lake Durowskie was observed at the end of the twentieth and the beginning of the twenty-first century. Significant nutrient loads were flowing to the lake with waters of the Struga Gołaniecka River from the catchment area. The total lake catchment with an area of 236.1 km^2^ is covered mainly by fields, from which fertilizers penetrated the surface waters. The direct catchment area adjacent to the lake reaches 1581.3 ha, 58.3% of which is farmland, 33.5% forests, and 8.2% urban areas (Gołdyn et al. 2013). Steep slopes near the lake shore favor overland flow, not only from the city but also from the forest (Klimaszyk et al. 2015). Deterioration of water quality in Lake Durowskie was manifested in cyanobacteria-dominated summer water blooms, oxygen depletion in the meta- and hypolimnion, and the presence of hydrogen sulfide in the hypolimnion up to 2008 (Gołdyn et al. 2013). Therefore, restoration treatments were initiated in 2009 to improve the lake water quality using three restoration methods, i.e., (i) hypolimnion aeration by wind-driven pulverizing aerators, (ii) P inactivation in the water column using low doses of iron sulfate and magnesium chloride, and (iii) biomanipulation, with predatory fish fry stocking. These methods, in accordance with the concept of sustainable restoration, are energy-efficient and not destructive for the most of the biota (Gołdyn et al. 2014). Hypolimnetic aeration is a method that uses wind energy to rise water overlying the sediments and after oxygenating it on the surface of the lake, draining it again to the bottom zone without destroying the thermal stratification (Podsiadłowski et al. 2018). The purpose of the iron treatment was to precipitate P from the water column to the bottom sediments. Small doses of chemicals, i.e., 4–15 kg of iron sulfide per hectare, do not coagulate the suspended solids from the water column but adsorb and bind phosphorus. The treatment was repeated 3–5 times during the vegetation season, eliminating P from the water column. Biomanipulation was based on pike and pikeperch fry stocking to increase the contribution of predatory fish in the lake ichthyofauna. Pike stocking was conducted irregularly, with a greater quantity of fry in 2011, i.e., 100,000 ind. In the case of pikeperch fry, the highest amounts were introduced in 2010, namely 114,000 ind.

## Material and Methods

**Fig. 1:**
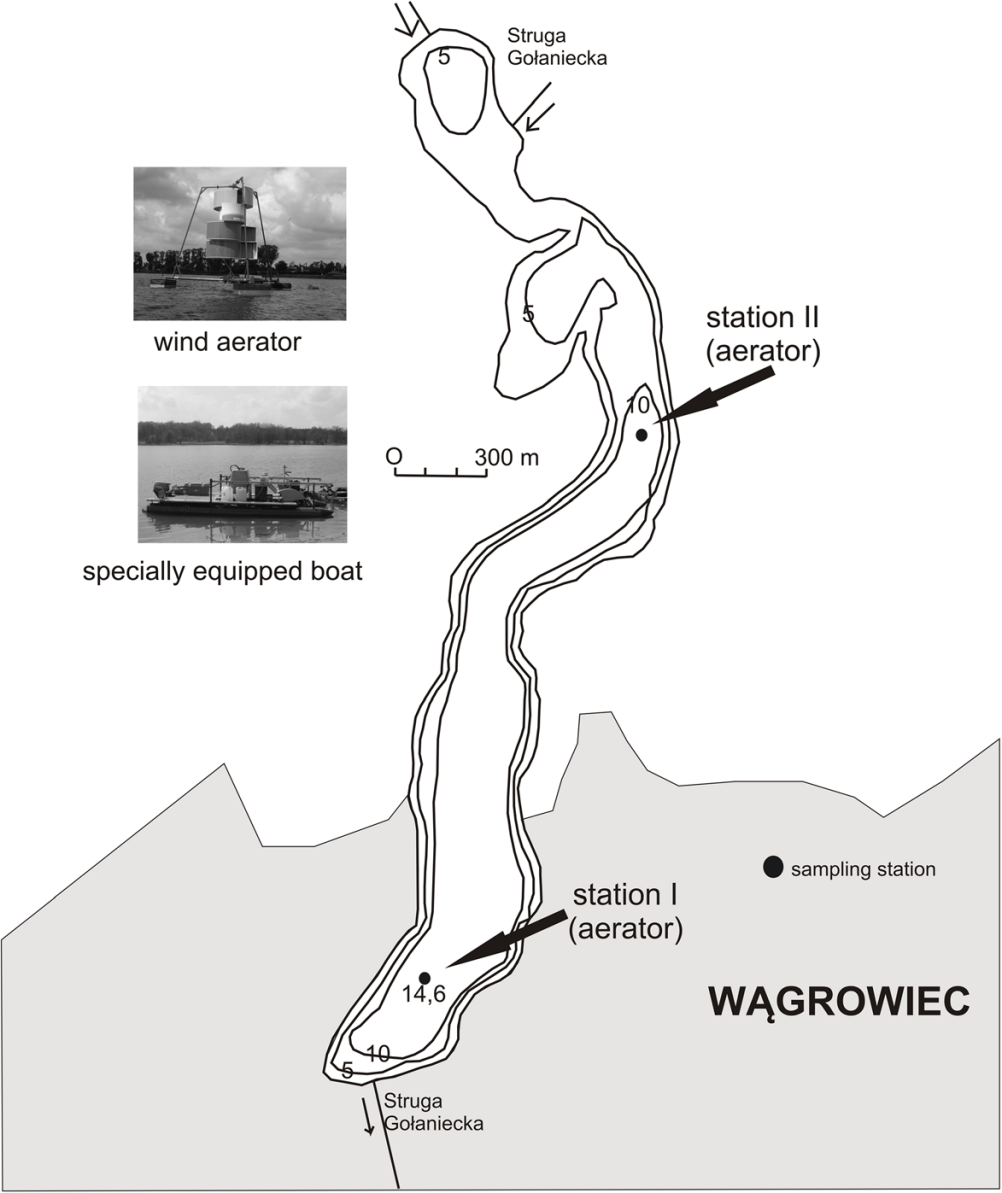
Location of the sampling stations on a bathymetric map of Lake Durowskie (after Gołdyn et al. 2013, with modification)

Research on Lake Durowskie sediments began in 2009 when the restoration started, and continued until 2016. Sediments were sampled at two stations; station I was located at the deepest place of the lake, i.e., at a depth of 14.6 m in the southern part of the lake, and station II in the northern part at a depth of 12 m. Both stations were localized in the vicinity of pulverizing aerators (Fig. 1). Samples from the surface sediment layer were collected every month between February and December each year using a Kajak core sampler.

The collected samples were analyzed for total phosphorus (TP) concentration and its individual extractable P fractions based on an analytical pattern proposed by Psenner et al. (1988) in modification by Lewandowski et al. (2003). The following were assayed in a volume of 1 cm^3^ of wet sediment: loosely bound phosphorus (NH_4_Cl-P), extraction with 1 M NH_4_Cl for 2 h; phosphorus bound with iron (BD-P), extraction with a mixture (1:1) of 0.11 M NaHCO_3_ and 0.11 M Na_2_S_2_O_4_ for 2 h; phosphorus bound with aluminum (NaOH-P) and organic matter (NaOH-NRP), extraction with 1.0 M NaOH for 18 h; phosphorus bound with calcium (HCl-P), extraction with 0.5 M HCl for 18 h and the residue (Res-P), being the difference between TP concentration and the sum of the first four fractions.

Sediment samples were also analyzed for organic matter content (%) by drying to constant weight and incineration at 550 °C. Water content (%) was calculated from the difference between the wet and dry weight of the sample.

The content of the following parameters: nitrogen, sulfates, iron, calcium, and magnesium was determined in bottom sediments from both stations as well. Total nitrogen was analyzed using a TOC-L Shimadzu analyzer with a TNM-L unit via catalytic thermal decomposition and chemiluminescence methods (Shimadzu, Japan). SO_4_^2−^ was analyzed using the gravimetric method. Total water hardness and Ca^2+^ concentration were determined by the versenate method, while Mg^2+^ concentration was calculated from the difference between total hardness and the concentration of Ca^2+^ ions (Standard Methods 1999). Determination of total Fe was made using atomic absorption spectrometry with flame atomization (F-AAS) (Shimadzu AA7000 Japan).

Soluble reactive phosphorus (SRP) and TP concentrations were also analyzed in water overlying the sediments (collected in situ) and in interstitial water (after sediment centrifugation for 1 h at 3000 rpm).

**Fig. 2:**
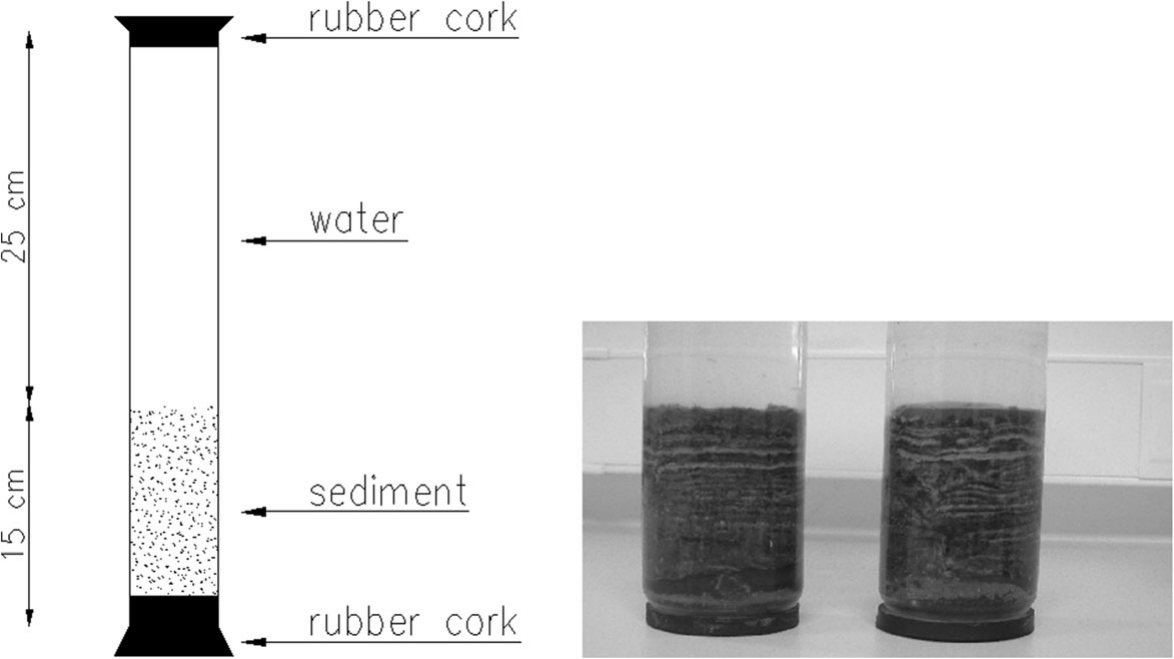
Scheme and photo of tubes with intact sediment cores and overlying water sampled in Lake Durowskie (annual lamination is visible)

The exchange of P in the sediment-water interphase was determined in the course of ex situ experiments with the use of intact sediment cores, collected by a modified core sediment sampler at both research stations in triplicates. Transparent tubes with ca 15 cm of sediments and overlying water were closed with rubber stoppers and transported to the laboratory (Fig. 2). Cores were sampled seasonally, four times a year.

Sediment cores were exposed in laboratory conditions in darkness for 2 weeks at temperature and oxygen concentrations similar to those occurring naturally in the lake hypolimnion at the time of sampling. The tubes remained closed with rubber stoppers throughout the experiment in the case of anaerobic conditions noted near the lake bottom. Artificial aeration was applied when oxygen content decreased significantly in comparison to initial conditions. Water samples were collected every 2–3 days from each core for the spectrophotometrical analysis of TP, with ascorbic acid as a reductor and replaced with water sampled from lake hypolimnion with an identified TP content. Changes in TP concentrations in water were calculated into internal P loading from 1 m^2^ of sediment per day, based on the differences of TP content between experiment days, the volume of water overlying the sediment core in the tube, and the area of sediment. Additionally, basic water features were measured prior to the sampling: temperature, oxygen concentration, pH, and conductivity, using a WTW Multi 350-i meter.

Statistical analyses were made with the STATISTICA software, version 10.0. To confirm the significance of differences between the analyzed sediment features in time and space, non-parametric tests were used, i.e., Kruskal-Wallis and Mann-Whitney *U* tests. The Pearson correlation coefficient was used to determine the relationship between the variables. To analyze the dependence of phosphorus release on the properties of bottom sediments, as well as interstitial and overlying water, the canonical RDA analysis from the Canoco for Windows 4.5 package was used (Ter Braak and Šmilauer 2002).

## Results

Lake Durowskie sediments were represented by gyttja sediments and characterized by a clear laminar structure, formed by dark and fair layers (Fig. 2).

### The Overlying Water

**Fig. 3:**
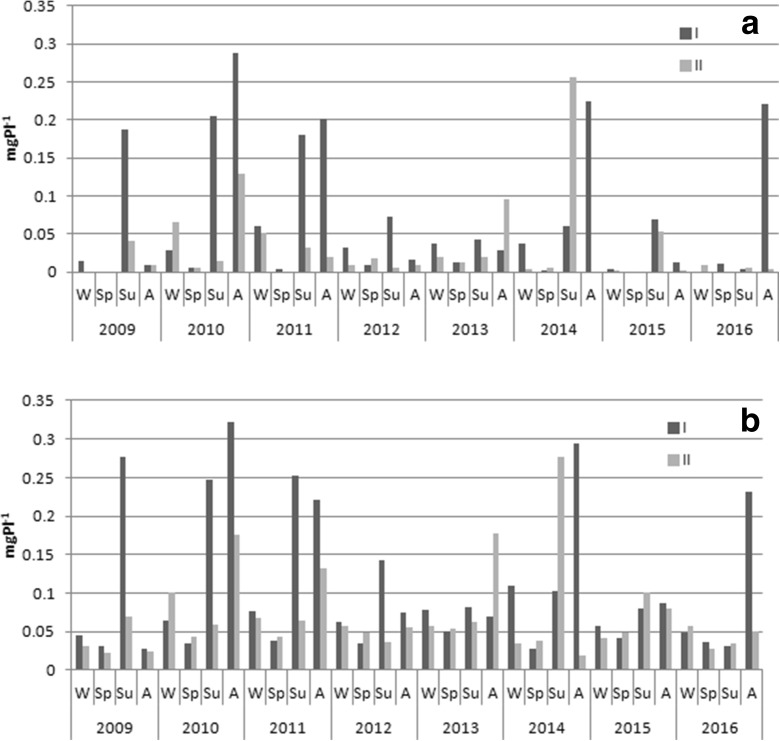
Changes of SRP (**a**) and TP (**b**) concentrations in the water overlying the sediments in particular seasons of 2009–2016 (W, winter; SP, spring; SU, summer; A, autumn)

SRP and TP concentrations in the overlying water changed both in terms of seasons and years. Higher values were observed in summer and autumn, reaching maximally 0.29 mgP l^−1^ at station I and 0.26 mgP l^−1^ at station II for SRP and 0.32 mgP l^−1^ and 0.28 mgP l^−1^ for TP, respectively. Concentrations below 0.1 mgP l^−1^ were noted in the other seasons (Fig. 3).

**Fig. 4:**
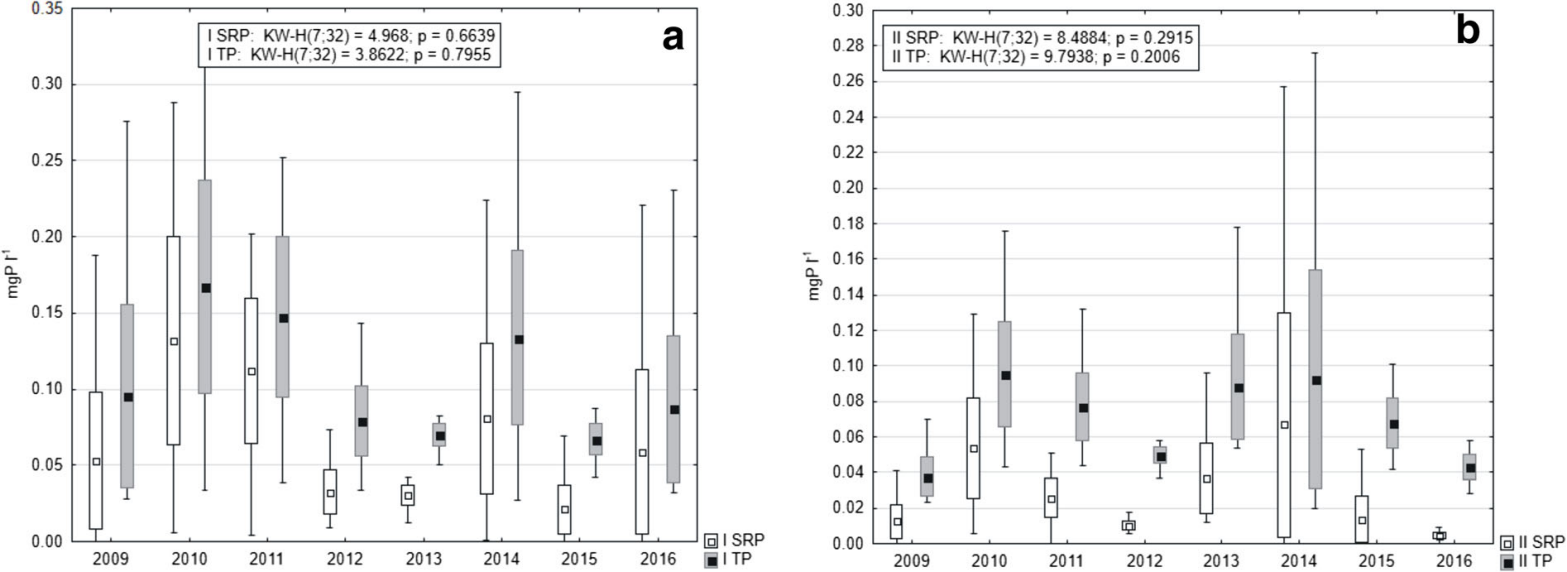
Changes of SRP and TP concentrations in water overlying the sediments in 2009–2016 at station I (**a**) and station II (**b**) (box, mean ± standard deviation; whiskers, minimum and maximum)

Mean concentrations of both P forms were higher at station I (Fig. 4). In the case of SRP, statistically significant differences were noted between the analyzed stations (Mann-Whitney *U* test, *U* = 361, *p* = 0.043, *z* = 2.02). Additionally, distinct fluctuations were observed in subsequent years. The highest contents at station I were noted in 2010, the lowest in 2013, while at station II in the years 2014 and 2016, respectively. Nevertheless, no statistical differences were stated between the years (Kruskal-Wallis test) at both stations (Fig. 4).

### Pore Water

**Fig. 5:**
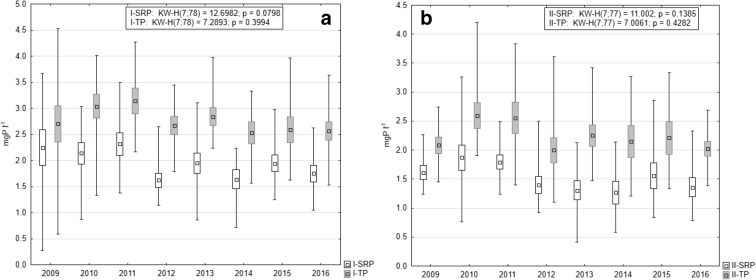
The changes of SRP and TP in pore water of Lake Durowskie sediments in 2009–2016 at station I (**a**) and station II (**b**) (box, mean ± standard deviation; whiskers, minimum and maximum)

SRP and TP concentrations in pore waters of Lake Durowskie were slightly higher at station I (Fig. 5) and this difference was statistically significant (Mann-Whitney *U* test for SRP: *U* = 1819, *p* = 0.000, *z* = 4.24 and for TP: *U* = 1660, *p* = 0.000, *z* = 4.80).

The highest mean annual content of P was noted at station I in 2011, up to 2.32 mgP l^−1^ for SRP and 3.14 mgP l^−1^ for TP, while in 2010 at station II, it was 1.87 mgP l^−1^ and 2.59 mgP l^−1^, respectively. Low values were observed in 2012 at both stations, i.e., 1.62 mgP l^−1^ and 2.67 mgP l^−1^ at station I and 1.39 mgP l^−1^ and 1.99 mgP l^−1^ at station II, for SRP and TP, respectively. These concentrations re-increased in succeeding years, but to values lower than noted at the beginning of the research (Fig. 5). Nevertheless, there were no statistical differences between the analyzed years (Kruskal-Wallis test).

### Total P in Sediments

**Fig. 6:**
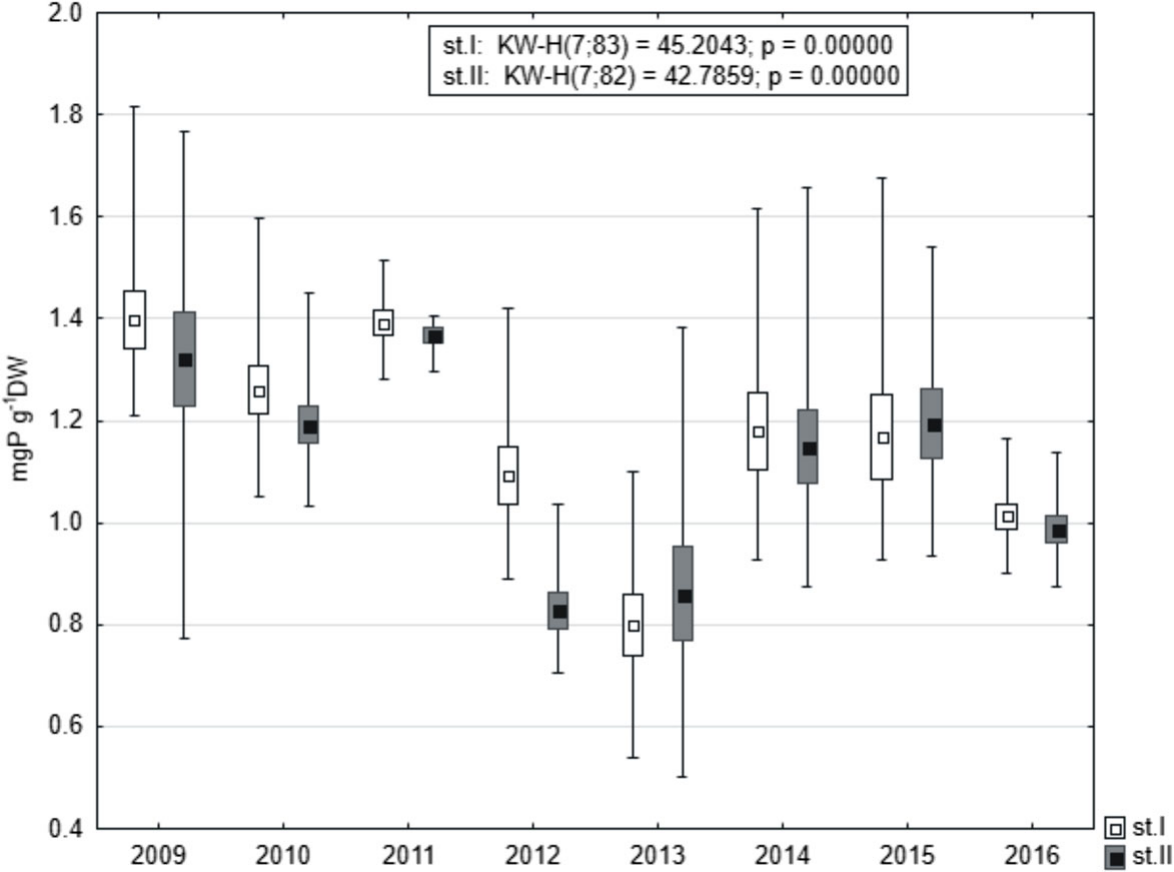
Concentrations of TP in bottom sediments of Lake Durowskie in 2009–2016 at two stations (box, mean ± standard deviation; whiskers, minimum and maximum)

Mean TP content in sediments was usually slightly higher at station I (Fig. 6); however, no statistical differences were revealed by the Mann-Whitney *U* test (*U* = 2997, *p* = 0.284, *z* = 1.07). Distinct fluctuations were observed between the analyzed years. The highest mean TP content at station I was noted in 2009 (1.39 mgP g^−1^ DW), while at station II in 2011 (1.37 mgP g^−1^ DW). Diminished values were stated in 2012–2013, i.e., below 0.79 mgP g^−1^ DW at station I and below 0.83 mgP g^−1^ DW at station II. A re-increase of TP in sediments appeared in 2014–2015, while in 2016 the values decreased again to about 1.0 mgP g^−1^ DW. Statistically significant differences between the analyzed years at both research stations were revealed by the Kruskal-Wallis test (Fig. 6).

### P Fractions in Sediments

Mean values as well as the contribution of P fractions to TP were similar at both research stations (Table 2); thus, no statistical differences were observed (Mann-Whitney *U* test, *p* > 0.05). The lowest content characterized NaOH-P, i.e., P related to aluminum (0.040 mgP g^−1^ DW at station I and 0.036 mgP g^−1^ DW at station II). Mean contribution of the three most mobile fractions (NH_4_Cl-P, BD-P, and NaOH-P) was quite low, reaching 6.28% at station I and 6.34% at station II. The highest share (over 45%) characterized the Res-P fraction, i.e., P permanently buried in sediments in organic and inorganic compounds (Table 2).

**Table 2:** Mean, minimum, and maximum values of P fractions content in sediments (in mgP g^−1^ DW) and its contribution (in %) to sediment TP in Lake Durowskie in 2009–2016 at both stations

Station	Fraction	Mean	Minimum	Maximum	SD	Number
I	NH_4_Cl-P	0.109 (9.61)	0.008 (0.81)	0.229 (18.98)	0.044 (3.65)	84
	BD-P	0.063 (5.51)	0.008 (0.81)	0.152 (16.63)	0.034 (3.01)	84
	NaOH-P	0.040 (3.74)	0 (0)	0.135 (12.34)	0.030 (2.93)	84
	NaOH-NRP	0.199 (17.28)	0.054 (5.90)	0.471 (41.15)	0.08 (6.63)	84
	HCl-P	0.212 (17.90)	0.041 (4.39)	0.407 (32.40)	0.096 (6.80)	84
	Res-P	0.534 (45.87)	0.111 (14.49)	1.182 (79.09)	0.212 (14.28)	84
II	NH_4_Cl-P	0.108 (9.97)	0.030 (3.09)	0.207 (19.89)	0.039 (3.48)	84
	BD-P	0.064 (5.66)	0.012 (1.20)	0.187 (13.54)	0.038 (2.98)	84
	NaOH-P	0.036 (3.39)	0 (0)	0.122 (11.51)	0.028 (2.56)	84
	NaOH-NRP	0.190 (16.81)	0.054 (5.17)	0.382 (30.04)	0.081 (5.98)	84
	HCl-P	0.194 (17.02)	0.031 (2.95)	0.391 (28.06)	0.093 (6.50)	84
	Res-P	0.532 (47.08)	0.171 (22.83)	1.008 (81.90)	0.191 (14.0)	84

**Fig. 7:**
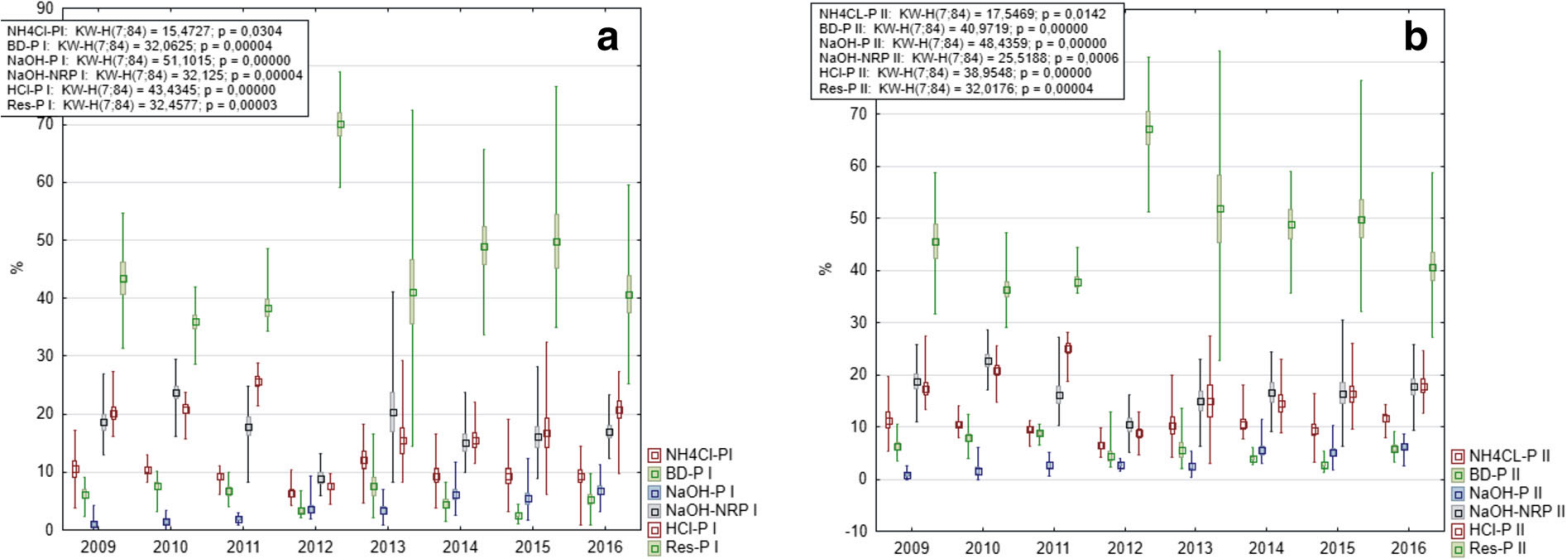
Changes of mean content of particular P fractions in Lake Durowskie sediments in 2009–2016 with the results of Kruskal-Wallis test at station I (**a**) and station II (**b**) (box, mean ± standard deviation; whiskers, minimum and maximum)

Temporal changes of P fraction content in sediment differed among the fractions. A decreasing trend was observed in the case of P related to iron (BD-P) as well as P bound to organic compounds and calcium (NaOH-NRP and HCl-P). A slight increase was noted for NaOH-P (P related to Al), while two other fractions, i.e., Res-P and NH_4_Cl-P revealed distinct fluctuations over time. Statistically significant differences were noted for all P fractions (Fig. 7).

### Other Sediment Characteristics

**Fig. 8:**
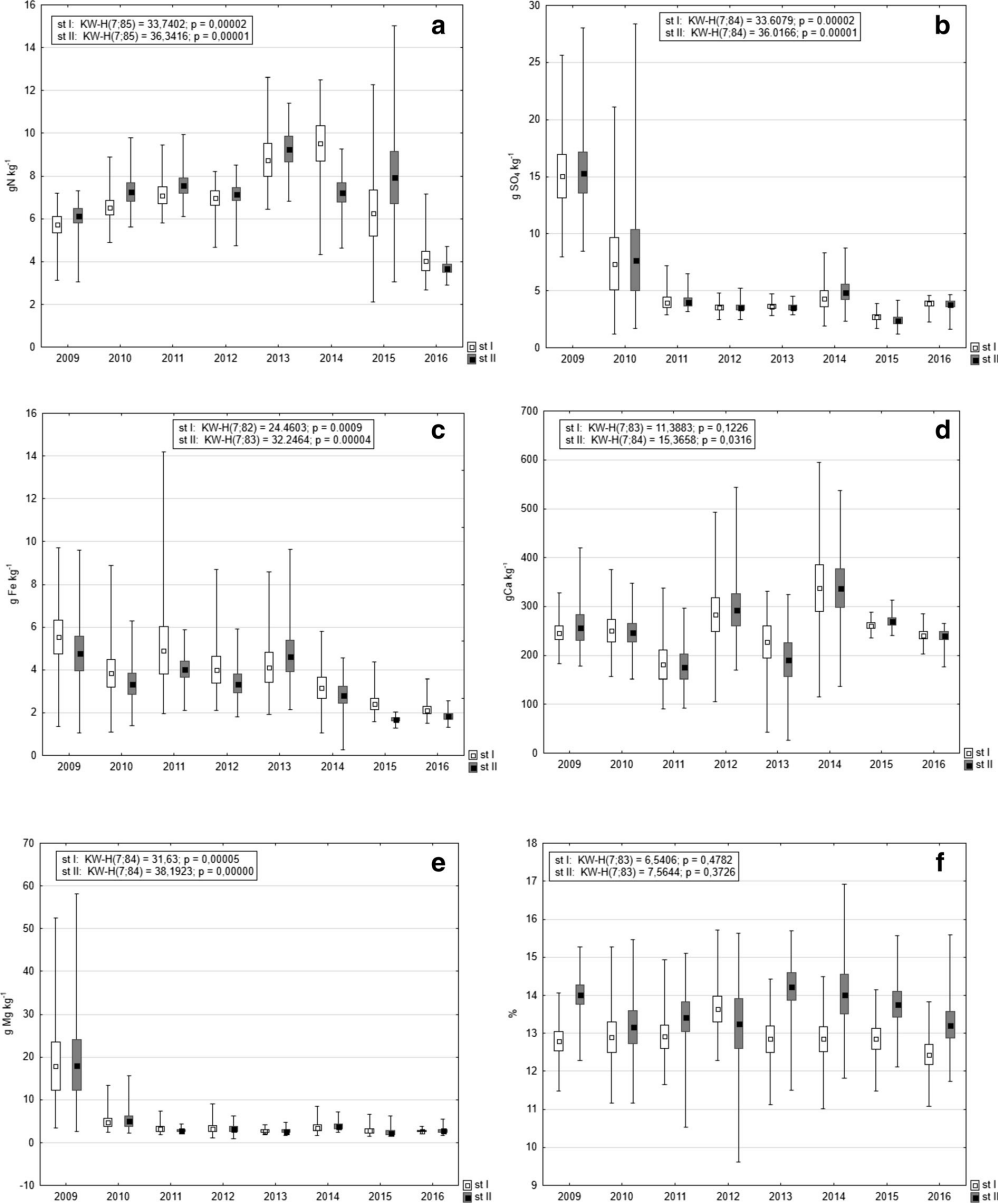
Comparison of mean values (squares inside the boxes) of nitrogen (**a**), sulfur (**b**), iron (**c**), calcium (**d**), magnesium (**e**), and organic matter (**f**) in the bottom sediments at both stations in the Durowskie Lake in 2009–2016 (box, mean ± standard deviation; whiskers, minimum and maximum)

Mean organic matter contribution to DW of sediments was slightly higher at station II (Table 4) and this difference between stations was statistically significant (Mann-Whitney *U* test: *U* = 2161.0, *p* = 0.000, *z* = − 4.14). Temporal changes of organic matter share were indiscernible and statistically insignificant. The highest value at station I was noted in 2012, while at station II in 2013 (Fig. 8f).

The Mann-Whitney *U* test revealed no statistically significant differences in nitrogen, iron, calcium, magnesium, and sulfur between lake stations (*p* > 0.05); however, some differences were observed between the years (Fig. 8). Total nitrogen in lake sediments was characterized by low fluctuations of content—the lowest values were noted in 2016 and the highest in 2014 (station I) and 2013 (station II). Mean content of sulfur shifted especially at the beginning of the research period. Its highest values were recorded in 2009 (ca 15 gSO_4_ kg^−1^), diminishing to 7 gSO_4_ kg^−1^ in 2010 and to 3–4 gSO_4_ kg^−1^ in successive years. The lowest sulfur content appeared in 2015–2.7 gSO_4_ kg^−1^ (station I) and 2.4 gSO_4_ kg^−1^ (station II) (Fig. 8b). In the case of iron concentrations, the variability of values was not as high as for sulfur. The maximum was observed in 2009 (5.6 gFe kg^−1^ at station I and 4.8 gFe kg^−1^ at station II), decreasing in the following years to 2.13 gFe kg^−1^ in 2016 and 1.7 gFe kg^−1^ in 2015 (Fig. 8c). Mean calcium content varied from ca 180 gCa kg^−1^ in 2011 to 330 gCa kg^−1^ in 2014. Statistically significant differences for this metal were noted only at station II (Fig. 8d). A decreasing temporal trend was noted for magnesium concentrations—from 18 gMg kg^−1^ in 2009 to 2.6 gMg kg^−1^ in 2013 (station I) and 2.3 gMg kg^−1^ in 2015 (station II) (Fig. 8e).

### Internal P Loading

Both spatial and temporal variability of internal P loading from the sediments revealed ex situ experiments. The data of measured water quality variables in the water overlying the cores of sediments is presented in Table 3.

**Table 3:** The mean values (± SD) of physical parameters (mean of 2009–2016) in water overlying the sediment cores (the average of the experiment) sampled in Lake Durowskie

Station	Season	Temperature (°C)		Conductivity (μS cm^−1^)		pH	Oxygen (mgO_2_ l^−1^)		Redox	
		Mean	SD	Mean	SD	Range	Mean	SD	Mean	SD
I	W	4.36	0.34	714	48.9	7.04–8.28	4.22	1.6	+ 186.7	53.2
	Sp	4.50	0.38	702	49.3	7.10–8.70	4.47	1.78	+ 158.8	67.6
	Su	4.91	0.72	712	59.6	7.01–7.86	0.31	0.32	− 182.8	74.8
	A	4.45	0.35	722	60.1	7.01–7.62	0.81	0.81	− 128.6	86.7
II	W	4.36	0.34	712	52.1	7.01–8.18	4.23	2.27	+ 188.3	58.4
	Sp	4.50	0.38	697	48.8	7.3–8.31	4.63	1.77	+ 138.1	88.4
	Su	4.91	0.72	697	52.8	7.02–7.65	1.03	1.23	− 138.6	33.3
	A	4.45	0.35	674	42.1	7.1–7.97	1.51	1.23	− 72.3	89.6

*W* winter, *SP* spring, *SU* summer, *A* autumn

**Fig. 9:**
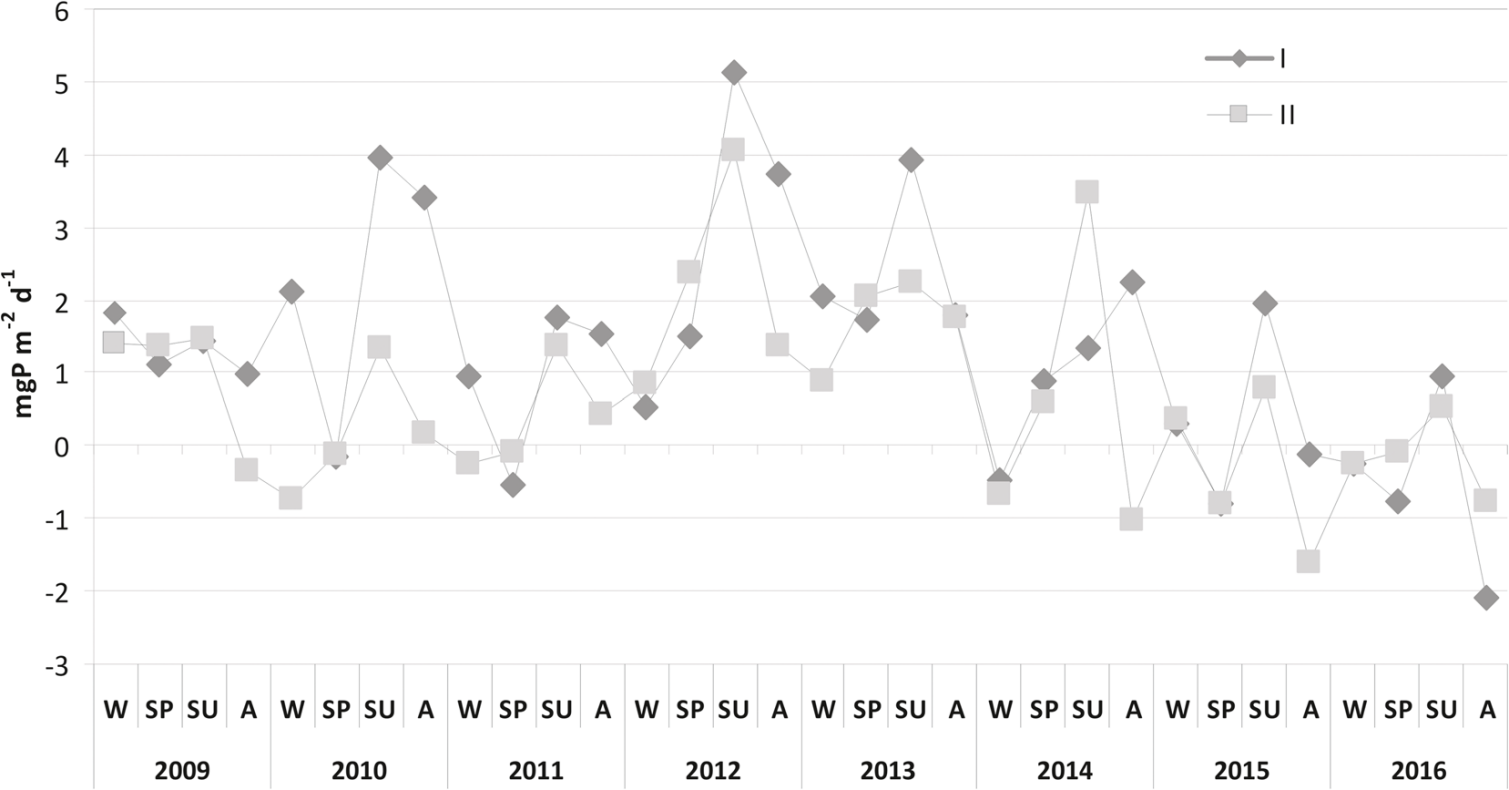
Seasonal changes of domination of phosphorus release (positive values) or accumulation in bottom sediments (negative values) in Lake Durowskie at both stations in 2009–2016 (W, winter; SP, spring; SU, summer; A, autumn)

A distinct domination of P release over its accumulation was observed in summer and autumn 2012, with maximum values of 5.12 mgP m^−2^ day^−1^ at station I and 4.08 mgP m^−2^ day^−1^ at station II. P loading did not exceed 4 mgP m^−2^ day^−1^ in all other seasons. The domination of P accumulation was noted in spring and winter as well as in autumn, reaching maximally 2.08 mgP m^−2^ day^−1^ in 2016 at station I (Fig. 9).

**Fig. 10:**
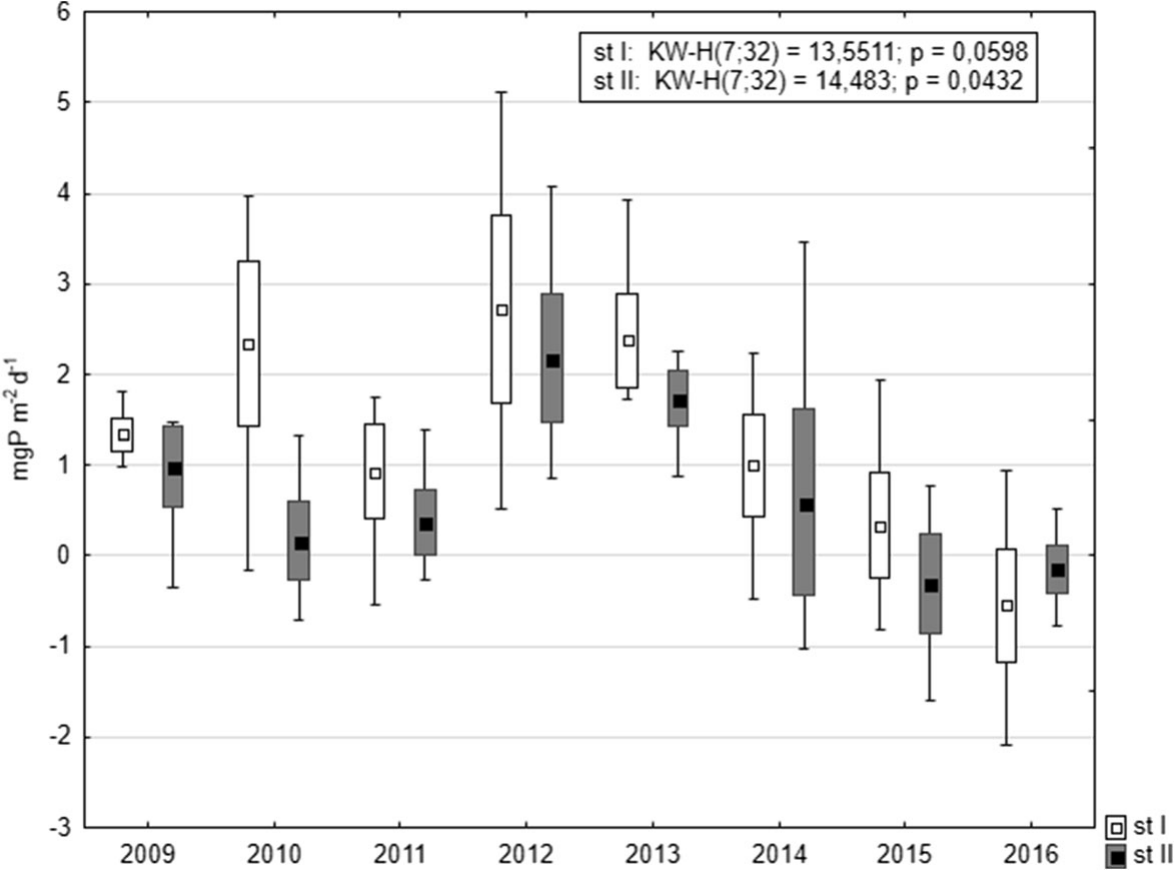
Comparison of mean annual values (squares inside the boxes) of phosphorus exchange across the sediment-water interphase at both stations in Lake Durowskie in 2009–2016 (box, mean ± standard deviation; whiskers, minimum and maximum)

Until 2015, mean annual P internal loading was higher at station I, although no statistically significant differences were stated between stations (Mann-Whitney *U* test: *U* = 382, *p* = 0.08, *z* = 1.73). This loading was the highest in 2012, i.e., 2.72 mgP m^−2^ day^−1^ and 2.17 mgP m^−2^ day^−1^ at stations I and II, respectively. A slight mean annual P accumulation appeared at station I in 2016 (0.54 mgP m^−2^ day^−1^), but it is worth emphasizing that at station II it had already been stated in 2015 and continued in 2016, although on a low level, i.e., 0.31 mgP m^−2^ day^−1^ and 0.15 mgP m^−2^ day^−1^, respectively. Changes observed between the analyzed years at station II were statistically significant (Fig. 10).

**Fig. 11:**
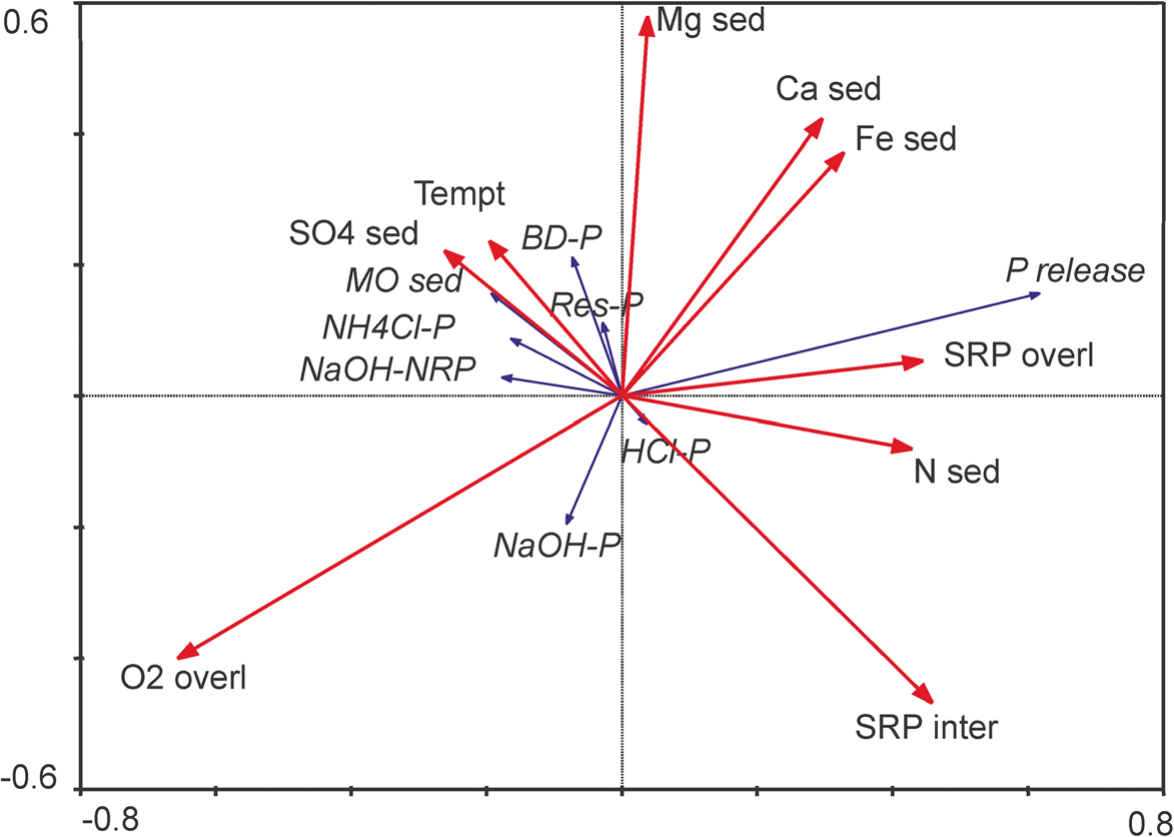
The results of the redundancy analysis (RDA) for dependence of P in bottom sediments on the characteristics of these sediments and overlying waters. Explanations: sed, in sediments; overl, in overlying water; inter, in interstitial water; MO, organic matter; Tempt, temperature

RDA analysis showed that the P release from bottom sediments is most strongly negatively related to the oxygen content in the overlying water (Fig. 11). It also depends on the SRP in the overlying water and to a lesser extent on the SRP in the interstitial water. However, it is not dependent on the temperature of the overlying water. P release depends also partly on the N content in the sediments, but not on the organic matter content, which is more related to the Res-P fraction.

## Discussion

The most commonly expected effect in lakes subjected to restoration treatments is the reduction or elimination of phytoplankton blooms caused by the large numbers and biomass of cyanobacteria, which are often toxic (Søndergaard et al. 2002; Kobos et al. 2013). Methods aiming at the limitation of available P by precipitation and immobilization in bottom sediments are the most commonly used in order to achieve this goal (Dunalska et al. 2015; Søndergaard et al. 2002; Zamparas and Zacharias 2014). Lake Durowskie is an example of the use of sustainable restoration, which was aimed at improving water quality by precise inactivation of SRP in the water column using small doses of chemicals, leading to the elimination of water blooms and retention P in bottom sediments. The biomanipulation method was particularly helpful in phytoplankton removal from the water column due to the increased food pressure exerted by zooplankton. In turn, oxygenation of the water overlying the sediment led to an increase in the redox potential of the sediment-water interphase and better retention of phosphorus on metal compounds (Søndergaard et al. 2002; Gołdyn et al. 2014). The use of such non-aggressive restoration methods slowly transforms the lake ecosystem, without causing drastic changes in the composition of the biota. Thanks to increasing the biodiversity various relationships are harnessed in the food web network to support the restoration process (Gołdyn et al. 2013; Rosińska et al. 2018).

The sediments of Lake Durowskie at the both analyzed stations have clearly marked annual lamination (Fig. 2). These laminae consist of bright spring-summer layers (most likely rich in calcium carbonate) and darker autumn-winter ones (created of detritus). This type of sediment is formed in dimictic lakes with oxygen depletion in the hypolimnion in summer, so that it is not subjected to bioturbation (Tylmann et al. 2013). The cells of planktonic algae and cyanobacteria in the pelagic zone, especially those belonging to autotrophic picoplankton serve as crystallization nuclei, indispensable in the precipitation of CaCO_3_ (Dittrich and Obst 2004; Szeląg-Wasielewska et al. 2015). The focus is shifted in the littoral zone towards macroscopic algae and vascular plants (Pełechaty et al. 2013). Due to the fact that the analyzed sediment was sampled in the pelagic zone, the plankton played a major role in their formation.

The release of phosphorus from bottom sediments can contribute up to 99% of the total P pool in shallow lakes (Boström et al. 1988; Jensen and Andersen 1992; Hullebusch et al. 2003). Thus, the intensity and duration of the internal loading may have a significant effect on the water quality (Jeppesen et al. 1991; Søndergaard et al. 1999). The process of internal P loading in Lake Durowskie was the most intense during summer periods at both research stations in all the analyzed years. This was related to the location of these stations within the profundal zone with oxygen deficits during thermal stratification (Gołdyn et al. 2013), which favors the release of phosphorus due to the iron reduction (Psenner 1984; Forsberg 1989). However, this released P was largely trapped in the water overlying the sediments and had little effect on the water quality in the surface water layer until the autumn mixing period. This was conducive to high P concentrations in the over-bottom water layer in summer and early autumn (Fig. 3). This was confirmed by the RDA analysis, which showed that P release was most strongly negatively related to the oxygen content as well as positively to the SRP in the overlying water. Although the process of P release from bottom sediments is highly influenced by the oxygen depletion in the sediment-water interface, it can also be observed in eutrophic waters in aerobic conditions (Boström et al. 1982; Kleeberg and Dudel 1997). This is common in shallow lakes where the temperature of bottom sediments increases in summer. This is promoted by intense mineralization of organic matter and rapid oxygen consumption in the sediment-water interphase. The resulting oxygen depletion leads to the reduction of metals and P release to the water column (Selig 2003; Golterman 2004; Kowalczewska-Madura et al. 2015). This increase in sediment temperature does not occur in the profundal zone of dimictic lakes. Therefore, the RDA analysis in Lake Durowskie did not show the dependence of P release on the temperature of overlying water. The temperature in water overlying the sediments fluctuated from 3.1 °C in winter to 8.4 °C in autumn (unpublished data). Nevertheless, P release prevailed over its accumulation in some winters and springs, especially at the beginning of the restoration process. However, this release was always much less than in summer. A smaller amount of organic matter of planktonic origin, settling to bottom sediments in the last years of the research resulted in lower oxygen consumption and good oxygenation of the water overlying the sediments occurring in winter and spring. It caused that the accumulation of phosphorus in the sediments prevailed its release. At greater depth full mixing of the water column in autumn is delayed and the sediments are worse oxygenated than in the shallower parts of the lake. Therefore, at the deeper station I, the phosphorus release was slightly higher than at the shallower station II (Table 4). However, there were no statistically significant differences between these data sets.

**Table 4:** Mean, minimum, maximum values, and SD of analyzed sediment characteristics in Lake Durowskie in 2009–2016

Station	Parameter	Unit	Mean	Minimum	Maximum	SD	Number
I	Phosphorus (bottom sediments)	mgP g^−1^ DW	1.17	0.54	1.81	0.25	83
II			1.11	0.50	1.76	0.26	83
I	Organic matter (bottom sediments)	%	12.89	11.02	15.71	1.03	83
II			13.63	9.61	16.92	1.37	83
I	SRP and TP (overlying water)	mgP l^−1^	0.064	0	0.29	0.08	32
I			0.106	0.027	0.323	0.09	32
II			0.028	0	0.26	0.05	32
II			0.069	0.020	0.276	0.05	32
I	SRP and TP (interstitial water)	mgP l^−1^	1.95	0.27	3.67	0.67	78
I			2.76	0.59	4.53	0.71	78
II			1.52	0.41	3.26	0.56	78
II			2.23	1.10	4.20	0.62	78
I	Phosphorus release or accumulation	mgP m^−2^ day^−1^	1.31	− 2.08	5.12	1.59	96
II			0.69	− 1.61	4.07	1.29	96
I	Nitrogen (bottom sediments)	gN kg^−1^	6.09	2.11	12.6	2.51	83
II			6.99	2.90	15.03	2.36	83
I	Sulfur (bottom sediments)	gSO_4_ kg^−1^	5.64	1.20	25.6	5.28	83
II			5.74	1.18	28.4	5.57	83
I	Iron (bottom sediments)	gFe kg^−1^	3.78	1.07	14.2	2.31	83
II			3.34	0.28	9.64	1.88	83
I	Calcium (bottom sediments)	gCa kg^−1^	254.1	42.5	594	93.9	83
II			252.1	25.9	544	95.05	83
I	Magnesium (bottom sediments)	gMg kg^−1^	5.17	1.17	52.6	8.43	83
II			5.17	1.0	58.1	8.68	83

Vertical P transport from the sediments to the epilimnion zone is a complex process that is only partially dependent on the morphometry of the lake (Mataraza and Cooke 1997). Osgood (1988) proposed a morphometric index, calculated as the ratio of mean depth ($\overline{z}$) to the square root of the surface area ($\sqrt{Ao}$), to estimate the duration of stratification and probability of mixing during summer storms. Lakes with an index of less than 6 or 7 are characterized by a shorter period of thermal stratification, often interrupted by strong summer storms, which promotes the transport of P from water overlying the sediments to the epilimnion (Straškraba et al. 1995). This index for Lake Durowskie is 6.61 but the arrangement of the longitudinal axis of the lake perpendicularly to the prevailing wind direction makes the stratification in the lake stable and long-lasting.

The analysis of the changes in the P release from bottom sediments in subsequent years revealed that during the first 4 years of restoration (2009–2012), the process was subject to significant fluctuations at both research stations. This is probably related to the variable hydrological conditions and the related differentiated external loading from the catchment area (Pociecha and Wilk-Woźniak 2006; Gołdyn et al. 2015). There was a significant relationship between the phosphorus concentration in the Durowskie Lake and supply of external phosphorus load by Struga Gołaniecka (the main inflow). Since 2012, only evident reduction of internal loading has been observed. This is not only related to the reduction of the P release intensity (especially during summer) but also to the increase of P accumulation in the bottom sediments. This accumulation did not exceed 1.0 mgP m^−2^ day^−1^ in the first 6 years of restoration and increased to over 2.0 mgP m^−2^ day^−1^ in the last 2 years of research (2015–2016). In addition, the dominance of phosphorus release from bottom sediments was noted only in summer of 2016, not exceeding 1.0 mgP m^−2^ day^−1^. Thus, the use of sustainable restoration increased the sorption complex of bottom sediments and caused the immobilization of phosphorus in bottom sediments, i.e., lower annual P internal loading than accumulation.

Changes in Lake Durowskie ecosystem related to the restoration treatments were also seen in the case of concentrations of SRP and TP in the pore waters. Phosphates in the interstitial water are in direct relation with the water overlying the sediment because crossing the solid-liquid boundary and releasing to the water column occurs by diffusion due to the difference in concentrations (Boström et al. 1982; Löfgren and Ryding 1985). Phosphorus concentration in the interstitial and overlying water allows us to predict the P pool potentially available for release from the bottom sediments (Psenner 1984; Søndergaard et al. 2002). However, RDA analysis showed that in Lake Durowskie SRP in interstitial and overlying water was dependent on each other, but not very closely. It was probably related to the fact that the correlation between the amount of internal P loading and the concentration of TP in interstitial water was statistically significant only in the case of station I (*r* = 0.353, *p* < 0.05).

On the other hand, in the case of SRP and TP concentrations in the overlying waters, there were no statistically significant differences between the analyzed years. There was, however, a correlation between the release of P from the bottom sediments and the concentration of TP in the bottom sediment at both analyzed stations (*r* = 0.43 for station I and *r* = 0.37 for station II, at *p* < 0.05).

Distinct changes during the study period were observed for the total P content and its fractions in bottom sediments. The content of P in sediments varied widely in the first years of restoration (2009–2012), similarly to the case of P release from bottom sediments. The amount of TP in the sediment decreased in periods when the release of P from bottom sediments clearly outweighed its accumulation. It was only when the trend to diminish the internal loading was fixed that the concentration of P in the sediment increased. In the case of station II, a statistically significant negative correlation between P release and TP content (*r* = − 0.39, *p* < 0.05) was noted.

Changes in TP concentration in bottom sediments were related to changes in its individual fractions. Analysis of quantitative ratios of fractions characterized by different bioavailability is a very important source of information on the persistence of phosphorus accumulation in sediments and the potential for its release into the water column (Kentzer 2001; Kaiserli et al. 2002; Pardo et al. 2003). When assessing bottom sediments as a potential source of P, its biological availability may be more important than the total content of this element in sediments (Psenner 1984; Kentzer 2001). The most bioavailable fractions (NH_4_Cl-P, BD-P, and NaOH-P) represented a small share of TP content (about 6%), which is a positive feature of this lake sediment. There were positive relationships between the content of the individual fractions and the concentration of TP data (*p* < 0.05) at both stations. Only the NaOH-P fraction was negatively correlated with total P (*r* = − 0.253). The relationship between P release and the content of NaOH-P (phosphorus fraction associated with metal oxides, mainly aluminum) was also negative (*r* = − 0.378, *p* < 0.05). This fraction is used to evaluate the short-term availability of P in sediments (Kozerski and Kleeberg 1998; Kentzer 2001) and therefore is considered a fraction available for algae (Zhou et al. 2001). While aluminum-related P is practically insoluble in the pH range that characterizes most natural waters, its release may, however, be a result of organic matter decomposition (Ting and Appan 1996). The content of this fraction was characterized by the smallest share in TP, as Al coagulants were not used in the restoration process.

A significant negative correlation at both stations was revealed between the total share of the three most mobile TP fractions (NH_4_Cl-P, BD-P, and NaOH-P) and the Res-P fraction, i.e., practically insoluble mineral and organic compounds (Psenner 1984) (*r* = − 0.73 and *r* = − 0.84, respectively). During the peak of P release (2012), the total contribution of the most mobile fractions decreased and, simultaneously, the Res-P fraction increased its share. Comparing the total share of the most mobile fractions and the Res-P fraction together with the change of P release/accumulation in the bottom sediments, it can be concluded that in recent years, when the decrease of internal P loading was observed, the fraction of NH_4_Cl-P, BD-P, and NaOH-P slightly increased and the Res-P fraction stabilized at a similar level. This may be related to the better oxygenation of the sediment-water interphase and the increasing sorption capacity of the sediments.

The NH_4_Cl-P and BD-P fractions also showed a positive relationship between SRP and TP concentrations in interstitial waters (*p* < 0.05), which resulted from the equilibrium between P adsorbed and dissolved in pore water. The low content of the BD-P fraction in Lake Durowskie sediments (about 5.5%) is related to the presence of periodic oxygen deficits at the analyzed research stations during summer. The oxygen depletion promotes iron reduction and the release of adsorbed phosphorus into the water column (Kenzer 2001; Nürnberg 2009). A positive relationship was found between the BD-P fraction and iron concentration in bottom sediments (*r* = 0.277, *p* < 0.05) in Lake Durowskie, which indicates the role of iron in P accumulation in sediments (Søndergaard et al. 2002). The content of iron in the sediments was small and amounted to 3.5 gFe kg^−1^ on average, despite the use of iron treatment. The relationship between iron and calcium (Fe/Ca) content in bottom sediments is used to assess the trophic conditions (Borówka 2007). This was very low in Lake Durowskie and amounted to 0.014, which was associated with periodic oxygen depletion of bottom sediments in summer. Low iron content in the bottom sediments of this lake resulted in ineffective P binding, as expected. The Fe/P ratio in the bottom sediments of Lake Durowskie was similar at both research stations and averaged 3.12. The release of soluble phosphorus was negatively correlated with the surface sediment Fe/P ratio. When the Fe/P ratio is above 15 (by weight), it may be able to control internal P loading by keeping the surface sediment oxidized (Jensen et al. 1992). Thus, in the studied lake, the iron content does not determine effective P binding.

The fraction containing almost half of the TP accumulated in the sediments was the Res-P fraction. The next two fractions had a similar proportion of P, i.e., the fraction associated with calcium and magnesium HCl-P and the fraction associated with the organic matter NaOH-NRP. High contribution of HCl-P and Res-P indicate the persistence of P accumulation in bottom sediments, whereas the NaOH-NRP fraction may be released due to the mineralization of organic matter (Mieszczakin and Wisniewski 2006). However, most of the organic matter in the sediments was hardly decomposable, represented by Res-P. Therefore, RDA analysis showed that the content of organic matter in sediments is correlated with Res-P, but not with P release. On the other hand, P release according to RDA analysis is dependent on the content of N in sediments, which is related to the presence of fresh organic matter with a high content of proteins, and thus easily decomposed organic matter. A similar contribution of P fractions was found in Lake Swarzędzkie and Lake Uzarzewskie (Kowalczewska-Madura et al. 2005, 2015, 2017).

Lake Durowskie sediments were characterized by high calcium content (on average over 250 g Ca kg^−1^). Calcium is one of the basic elements in the bottom sediments of many lakes. Such a high Ca content can be attributed to the dominance of ground water supply (Borówka 2007). Calcium-bound P is not sensitive to redox potential and once deposited in bottom sediments it may be stored there for a long time (Kufel et al. 2013). Significant calcium content in bottom sediments, as well as aluminum and iron, is a positive feature from the point of view of limiting internal P loading (Golterman et al. 1998). The high content of calcium and HCl-P fraction in the bottom sediments of Lake Durowskie should favor P binding in sediments.

Hydrogen sulfide was found in the hypolimnion during summer seasons as a product of bacterial sulfate decomposition that can block Fe^+2^ as an insoluble sulfide. SO_4_^2−^ may exert an influence in the P availability in the sediment due to the competition with o-P for anion sorption sites and H_2_S from SO_4_^2−^ reduction may bind to iron in anoxic sediments. Additionally, P mobilization and SO_4_^2−^ reduction in anoxic sediments generates a pH increase that would inhibit P sorption (Clavero et al. 1997). This mechanism explains the significant reduction of sulfate content in bottom sediments in the first 2 years of restoration. In the following years, their content was similar. Activation of the aerators caused the oxygen to enter the deepest lake zone and stimulated the intensive decomposition of organic matter by bacteria. Oxygen is consumed in the overlying waters, decomposing the sulfates, which irreversibly inhibit Fe^+2^, creating insoluble sulfide (Clavero et al. 1997).

As stated by Kleeberg et al. (2013), efficient P removal, irrespective of oxygen supply, is guaranteed by the release of sedimentary Fe and P in a ratio at which they will co-precipitate in the water column. Hypolimnetic P accumulation is thus insignificant for the epilimnetic P supply as the natural oxygenation during autumn mixing removes most of the P from the water column. Deppe and Benndorf (2002) suggested that in-lake dosage of iron compounds is regarded as an appropriate measure of eutrophication control, and Fe^2+^ salts may prove advantageous in several respects in comparison to Fe^3+^ compounds. Iron products should be preferred when redox conditions near the sediment allow a Fe application.

The P released from the bottom sediments of Lake Durowskie up to 5.12 mgP m^−2^ day^−1^ was rather low in comparison with other lakes subjected to restoration. It reached 13.02 mgP m^−2^ day^−1^ in Lake Uzarzewskie (Kowalczewska-Madura et al. 2017) and even 29.2 mgP m^−2^ day^−1^ in Lake Swarzędzkie (unpublished data) during their restoration. The Rusałka Reservoir achieved much more, namely 48.6 mgP m^−2^ day^−1^ (Kowalczewska-Madura et al. 2011). Lower values were observed in the mesoeutrophic Lake Strzeszyńskie, i.e., 2.8 mgP m^−2^ day^−1^ (Kowalczewska-Madura et al. 2015). The results of internal P loading from the last 2 years of Lake Durowskie research, lying between − 2 and + 2 mgP m^−2^ day^−1^, can therefore indicate conditions typical for mesotrophic lakes.

The research showed that for the first 8 years, since the introduction of a sustainable restoration of Lake Durowskie, the internal P loading from the bottom sediments changed, both in the seasonal aspect and in the subsequent years of research. There was a decrease of internal P loading from the bottom sediments to the overlying waters, while at the same time the accumulation of this element in sediments increased. The obtained results confirm the effectiveness of the treatment using low-dose P-binding chemicals together with supporting sustainable restoration methods, i.e., oxygenation of deep waters by a wind-driven aerator and biomanipulation. The supply of nutrient loads to the lake from the inflowing water of the main tributary the Struga Gołaniecka River means that the restoration cannot be completed until the quality of the upstream lakes is improved.
